# Virtual Surgical Planning (VSP) in Orthognathic Surgery for Non-Syndromic Cleft Patients: A Scoping Review of Trends and Clinical Outcomes

**DOI:** 10.3390/jcm15020911

**Published:** 2026-01-22

**Authors:** Jacek Drążek, Filip Bliźniak, Karolina Lubecka, Joanna Wołoszyn, Mateusz Kęska, Maciej Chęciński, Mariusz Szuta, Maciej Sikora

**Affiliations:** 1 Department of Maxillofacial Surgery, Rydygier Hospital, os. Złotej Jesieni 1, 31-826 Kraków, Poland; jacekdrazek777@gmail.com; 2 Department of Oral Surgery, Preventive Medicine Center, Komorowskiego 12, 30-106 Kraków, Poland; lubeckarolina@gmail.com; 3 Dental Clinic Outpatient Care Center, University Dental Clinic in Kraków, Montelupich 4, 31-155 Kraków, Poland; joannawoloszyn234@gmail.com; 4 Faculty of Medicine, Medical College, Jagiellonian University, Świętej Anny 12, 33-332 Kraków, Poland; matteuszkeska@gmail.com; 5 National Medical Institute of the Ministry of Interior and Administration, Wołoska 137 Str., 02-507 Warsaw, Poland; maciej.checinski@pimmswia.gov.pl (M.C.); sikora-maciej@wp.pl (M.S.); 6 Department of Maxillofacial Surgery, Hospital of the Ministry of Interior, Wojska Polskiego 51, 25-375 Kielce, Poland; 7 Chair of Oral Surgery, Medical College, Jagiellonian University, Montelupich 4, 31-155 Kraków, Poland; m.szuta@wp.pl; 8 Department of Biochemistry and Medical Chemistry, Pomeranian Medical University, Powstańców Wielkopolskich 72, 70-111 Szczecin, Poland

**Keywords:** cleft lip, cleft palate, computer-assisted surgery, orthognathic surgical procedures, oral and maxillofacial surgery, osteotomy

## Abstract

**Background/Objectives:** Isolated cleft lips and/or palates often require orthognathic treatment. Traditional planning based on 2D images and plaster models limits precision; therefore, virtual surgical planning (VSP) and Computer-Aided Design and Computer-Aided Manufacturing (CAD/CAM) technologies are increasingly being used. The aim of this scoping review was to analyze the techniques, outcomes, and gaps in research on VSP in orthognathics for patients with isolated (non-syndromic) clefts. **Methods:** Searches were conducted in July 2025 in seven databases (including PubMed, Scopus, and Cochrane) without language restrictions, in accordance with the PRISMA guidelines for scoping reviews. Of the 2836 records, 36 publications were eligible after deduplication and full-text screening, and their Level of Evidence (LoE) was assessed using the Oxford CEBM scale. A risk of bias assessment was also conducted according to JBI tools. **Results:** The identified studies primarily comprised LoE III and IV; there were no systematic reviews or randomized controlled trials (LoE I). Descriptions of bimaxillary procedures and LeFort I osteotomies dominated. The most commonly used software was ProPlan CMF, Dolphin 3D, and Rhinoceros, although other tools have emerged in recent years. The available studies suggest that VSP increases translational and rotational accuracy and facilitates individualized treatment, and bimaxillary procedures bring better functional and aesthetic outcomes in patients with severe maxillary hypoplasia. **Conclusions:** Despite the growing interest in VSP in orthognathics, the scientific evidence is limited and mostly of lower quality. Well-designed prospective studies are needed to assess the long-term stability, quality of life, and cost-effectiveness of modern technologies.

## 1. Introduction

Cleft lip and/or palate (CLP) is one of the most common congenital craniofacial anomalies, leading to complex dentofacial deformities that often require surgical intervention in the form of orthognathic surgery [1]. It can occur in patients as isolated defects or be associated with existing syndromes, such as Treacher Collins or Pierre Robin syndrome [2]. The etiology and treatment of syndromic and nonsyndromic clefts differ. These branches often develop separately and in separate treatment centers, each appropriately equipped for specific surgical procedures [3,4]. Traditionally, planning for such procedures relied on two-dimensional (2D) imaging and plaster models. It limited precision and the ability to predict outcomes [5]. It also led to imperfect results and certain deviations from the metric standards adopted before the procedure [6].

This is where the progress in the development of virtual, three-dimensional (3D) orthognathic surgery planning, visible in recent years, comes from. It results in enabling a more precise and individualized approach for each patient [7]. Techniques such as computer-assisted surgical planning (CASP), stereophotogrammetry, and precisely analyzed computed tomography (CT)—among others, cone beam computed tomography (CBCT)—allow for a more accurate anatomical representation and improved prediction of surgical outcomes [8,9]. Studies have shown that these approaches can lead to better aesthetic and functional results, as well as a higher patient satisfaction [10,11]. There is therefore not only a measurable benefit in the form of better postoperative effects related to a better spatial matching of the models used for a given patient, but also an increased quality of life (QoL) for people who have undergone this type of surgery [12]. There are also growing calls for expanding virtual planning of craniofacial surgical procedures to include the use of mixed reality (MR) or augmented reality (AR) glasses during the actual procedure [13]. This could elevate procedural accuracy to a whole new level, allowing the operator to virtually apply precise measurements and cutting lines to the patient’s tissues, without distortions caused by the inaccuracies of the physical materials used [14]. The extended benefits of using virtual, 3D surgery planning are the reduced costs of overall patient care, such as hospitalization time and potential complication management. Patients gain not only from a reduced treatment time but also from the reduced risk of unsatisfactory results compared to only two-dimensional surgery planning.

Despite the growing popularity of virtual orthognathic surgery planning in cleft patients, this topic has not yet received a scoping review that would define the current trends and outcomes of such procedures. The existing papers have focused primarily on general orthognathic surgery or have included heterogeneous cleft populations, limiting the ability to draw conclusions regarding isolated clefts. Furthermore, there is a lack of updated analyses that consider the latest digital planning technologies and their impact on outcomes in this specific subgroup of patients [15,16]. This topic is so emergent that it is worthwhile to systematically explore it and outline the potential directions in which future research and reviews should follow [17]. The objective of this scoping review is to systematically map, categorize, and synthesize the current evidence regarding VSP in orthognathic surgery for non-syndromic cleft patients (without other coexisting genetic syndromes, such as Van der Woude syndrome, Treacher Collins syndrome (mandibular dysostosis), Pierre Robin syndrome, Stickler syndrome, DiGeorge syndrome (22q11.2 microdeletion), Ehlers–Danlos syndrome, Opitz syndrome, Kabuki syndrome, and Smith–Lemli–Opitz syndrome).

## 2. Materials and Methods

### 2.1. Protocol and Registration

This study was designed and conducted as a scoping review, following the methodological framework provided by the Joanna Briggs Institute (JBI) and the PRISMA-ScR (Preferred Reporting Items for Systematic Reviews and Meta-Analyses extension for Scoping Reviews) guidelines [18,19,20]. The aim of this review was to systematically explore the available evidence on VSP software used in the orthognathic treatment of non-syndromic cleft patients, rather than to synthesize findings from randomized trials or perform meta-analyses. The process involved (1) defining the research question and eligibility criteria, (2) developing search strategies tailored to individual databases and sources of gray literature, (3) the systematic searching of selected databases, followed by deduplication and screening, (4) extracting key data relevant to software usage, clinical applications, and Level of Evidence (LoE) according to the Oxford Centre for Evidence-Based Medicine 2011 Levels of Evidence scale, (5) charting the data into structured tables and visual summaries to present the scope and distribution of current research, (6) synthesizing the results and presenting them in the form of appropriate maps and graphs, and (7) presenting the main results and conclusions of the work [21]. The protocol for this scoping review was prospectively registered in the Open Science Framework (OSF) on 20 July 2025, under the identifier osf.io/s2kzy [22].

### 2.2. Eligibility Criteria

Eligibility criteria were developed according to the PCC(COSTL) framework recommended by JBI for scoping reviews [23]. What this acronym stands for is explained in Table 1. Papers containing information on VSP for orthognathic surgery in patients with isolated CLP were included in the review. Papers describing this type of intervention in patients whose clefts were associated with the presence of genetic syndromes, such as Van der Woude syndrome, Treacher Collins syndrome (mandibular dysostosis), Pierre Robin syndrome, Stickler syndrome, DiGeorge syndrome (22q11.2 microdeletion), Ehlers–Danlos syndrome, Opitz syndrome, Kabuki syndrome, Smith–Lemli–Opitz syndrome, and similar syndromes, were excluded. This was due to the different etiology and treatment of these patients compared to nonsyndromic ones [24]. Animal, cadaver, and in vitro studies were not included in the review. Systematic reviews, meta-analyses, randomized controlled trials, non-randomized controlled trials, uncontrolled studies, case series, case reports, and master’s theses were included. Searches were not limited to English. The intervention had to be orthognathic surgery, previously planned virtually. Searches were not time-limited, with the final search being conducted on 22 July 2025.

### 2.3. Information Sources

The following databases and engines were searched: (1) Association for Computing Machinery: Guide to Computing Literature (The ACM Guide: over 3,900,000 records); (2) Bielefeld Academic Search Engine (BASE; over 400,000,000 records); (3) The Cochrane Library (Cochrane; over 2,000,000 records); (4) Embase (over 45,600,000 records); (5) National Library of Medicine: PubMed (PubMed; over 38,000,000 records); (6) Google Scholar (Scholar; over 160,000,000 records) (Table 2). No filters were used to maximize the accuracy of the study.

### 2.4. Search Strategy

The search strategy was designed. Its basic form was as follows: (orthognathic OR orthognathics OR fort) AND (vsp OR virtual OR casp OR computer OR dsw OR digital OR workflow OR 3dsp OR 3d-vsp OR cad OR cam OR vos OR dtp OR proplan OR planmeca OR romexis OR dolphin) AND (fissure OR split OR groove OR notch OR slit OR cleft OR disjunction OR disjunction).

The query was then matched to each of the searched databases and engines. The specific queries for each of them are available in Table A1.

### 2.5. Selection of Sources of Evidence

The retrieved records were first subjected to manual deduplication using the Rayyan automation tool (version 2025-07-23, Qatar Computing Research Institute, Doha, Qatar and Rayyan Systems, Cambridge, MA, USA). Two blinded investigators (J.W. and J.D.) then assessed the papers’ suitability for the study based solely on titles and abstracts. If the investigators’ assessments disagreed, the papers moved on to the next stage, full-text assessment. This was again performed by two investigators (J.W. and J.D.); in the event of disagreement, a third investigator (F.B.) had the casting voice.

### 2.6. Data Charting Process

Information regarding the study design was obtained independently from the source materials by two investigators (J.W. and J.D.) and standardized according to the Oxford Centre for Evidence-Based Medicine 2011 Levels of Evidence scale [21]. In cases of disagreement between the investigators, the third investigator (F.B.) had the final say. The adopted levels of evidence (LoEs) were as follows: (1) Level I—systematic reviews and meta-analyses of randomized controlled trials (RCTs), (2) Level II—lower-quality RCTs (e.g., with methodological limitations), (3) Level III—retrospective comparative studies, (4) Level IV—case series (with or without comparison group), and (5) Level V—expert opinions, narrative reviews, and cases.

### 2.7. Data Items

The most important data that were planned to be obtained included the type of orthognathic surgery, the software used to plan the procedure, and the LoE. The data were obtained in correlation with each other.

### 2.8. Critical Appraisal of Individual Sources of Evidence

A formal critical appraisal of the included studies was conducted using JBI standardized critical appraisal tools, applied according to the respective study design. Two reviewers independently assessed each study, and any disagreements were resolved through discussion until consensus was reached. The aim of this appraisal was not to exclude studies, but to provide a transparent evaluation of their methodological rigor and potential sources of bias. For records not meeting the criteria of peer-reviewed research articles (e.g., dissertations or theses), a risk of bias assessment was not performed, as the JBI tools cannot be appropriately applied to such formats. A summary of the risk of bias assessments is presented in the Supplementary Materials (Tables S1–S7).

### 2.9. Synthesis of Results

The results were tabulated and presented in graphs, highlighting the numerous relationships between the collected data. Bar charts and scatter plots were used to further illustrate the relationships and assumptions made when establishing the eligibility criteria. Data were tabulated using the Google Workspace package (version 2025-08-10, Google LLC, Mountain View, CA, USA).

## 3. Results

### 3.1. Selection of Sources of Evidence

**Figure 1:**
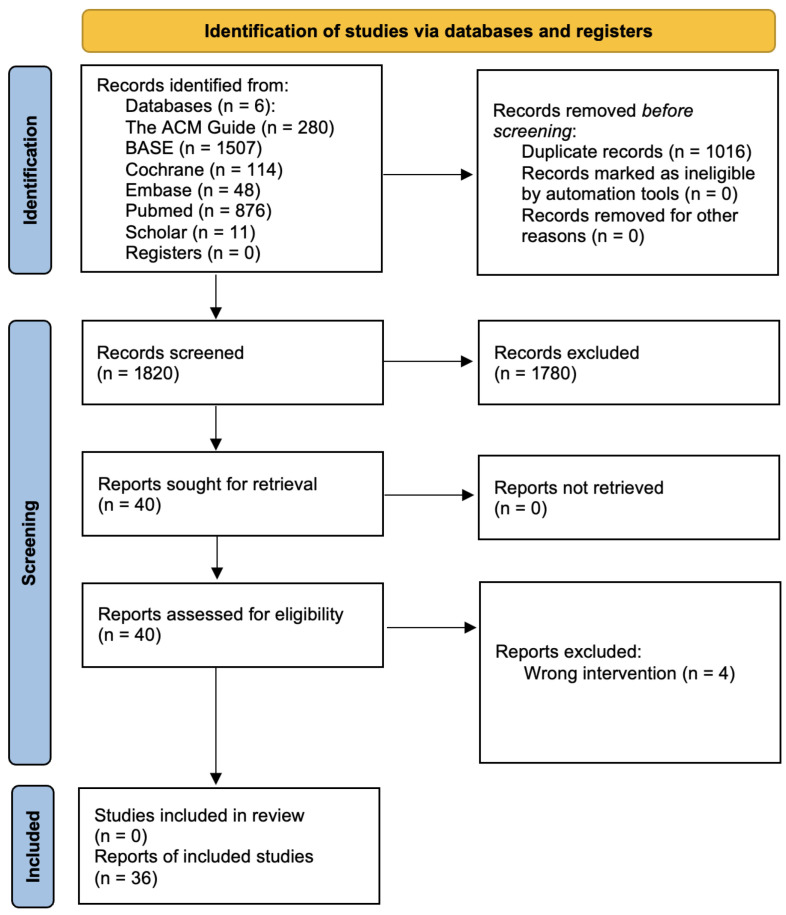
PRISMA flow diagram.

Of the 2836 records identified, 36 articles were ultimately qualified, meeting all the established criteria. This whole process is presented below using the PRISMA flow diagram (Figure 1). The LoEs were as follows: I (systematic reviews and meta-analyses of RCTs)—0 papers; II (lower-quality RCTs (e.g., with methodological limitations))—1 paper; III (retrospective comparative studies)—14 papers; IV (case series (with or without comparison group))—15 papers; and V (expert opinions, narrative reviews, cases)—6 papers. A high number of rejected articles during the screening stage can be attributed to the query designed to identify as many papers as possible in the databases. During the final selection phase, i.e., the full-text evaluation, four articles were rejected as not meeting the established eligibility criteria.

### 3.2. Characteristics of Sources of Evidence

**Figure 2:**
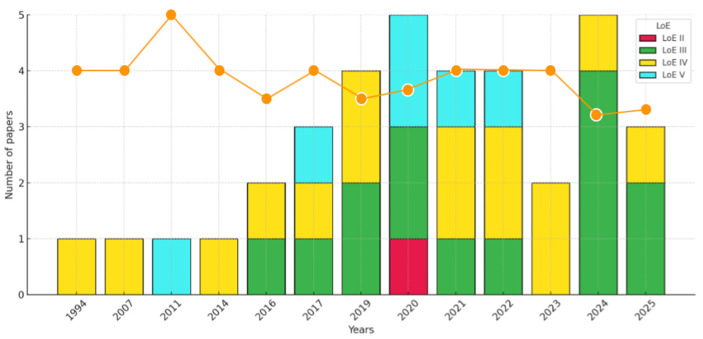
Distribution of the number of articles with different LoEs over time, with average LoE per year (orange line).

**Figure 3:**
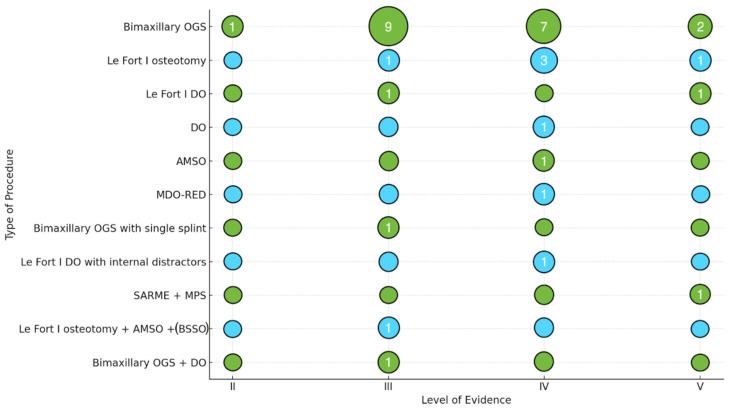
The number of papers relating to a given surgical procedure according to the LoE. Bimaxillary OGS—bimaxillary orthognathic surgery; DO—distraction osteogenesis; AMSO—anterior maxillary segmental osteotomy; MDO-RED—mandibular distraction osteogenesis using rigid external device; SARME—surgically assisted rapid maxillary expansion; MPS—maxillary posterior segmental osteotomy; BSSO—bilateral sagittal split osteotomy.

**Figure 4:**
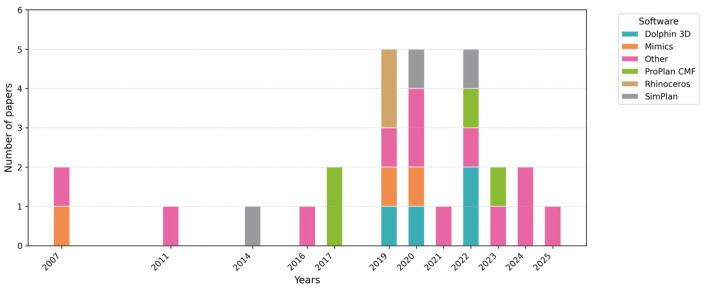
Popularity of VSP software over the years.

**Figure 5:**
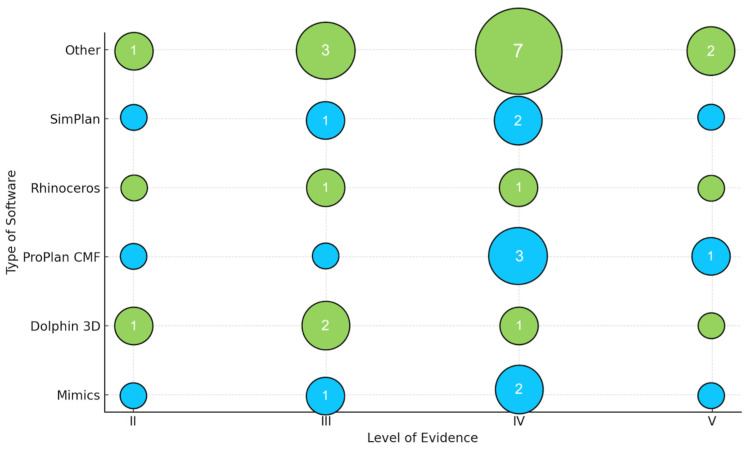
The number of papers relating to a given type of software according to the LoE.

For each included publication, data were compiled regarding (1) bibliographic information (first author, year, DOI), (2) the software used for the study design, (3) the type of surgical procedure performed, (4) the LoE, (5) clinical validation, (6) complications, (7) the follow-up time, and (8) the correlation of all these data with each other. These data are summarized in Figure 2, Figure 3, Figure 4 and Figure 5 and Table 3 and Table 4.

### 3.3. Critical Appraisal Within Sources of Evidence

Detailed study-level risk of bias assessments and extended methodological characteristics are provided in the Supplementary Materials (Tables S1–S7).

### 3.4. Results of Individual Sources of Evidence

In accordance with PRISMA-ScR guidelines, for each eligible evidence source, a set of data directly relevant to the questions and objectives of this review was presented. These data were extracted in a standardized manner using a data charting form, which included the following categories: (1) bibliographic information (author, publication year, DOI), (2) the software and virtual planning tools used (VSP/CAD-CAM), (3) the type of orthognathic procedure performed, (4) the LoE determined according to the Oxford Centre for Evidence-Based Medicine 2011 [21], (5) reported complications, (6) the postoperative follow-up time, and (7) other relevant clinical information, including the validation of results and description of treatment outcomes.

All these categories directly addressed the research questions, which led to the review covering (1) the types of software and virtual solutions used in planning, (2) the types and trends of orthognathic procedures performed in patients with isolated CLP, (3) the quality of scientific evidence, and (4) the claimed effectiveness, accuracy, and safety of VSP.

The data extraction results for individual studies are summarized in Table 3, which includes basic publication characteristics, including (1) the author and year, (2) the LoE, (3) the software used, (4) the postoperative course (complications), (5) the follow-up time.

Additional narrative and graphical presentations of the results from individual studies are provided in Figure 2, Figure 3, Figure 4 and Figure 5, which present (1) LoE trends over time, (2) the types of orthognathic procedures performed using VSP, (3) the popularity of selected digital planning software and tools, and (4) the LoE distribution by intervention type.

Detailed information about the software used in the studies is provided in Table 4, which includes data on (1) the number of studies using a specific system, (2) the scope of application of a given software, (3) the average accuracy of the procedure performance, (4) the advantages and limitations of selected platforms, and (5) the LoE and average sample sizes.

To maintain methodological transparency, a risk of bias assessment for individual clinical studies was performed using standard JBI tools. The results of this assessment are presented in the Supplementary Materials (Tables S1–S7), taking into account differences between study types (including cohort studies, cross-sectional studies, case series, quasi-experimental studies, and comparative studies). These tables provide a clear and objective summary of the quality and limitations of each study included in the review.

### 3.5. Synthesis of Results

In many reports and studies, IPS CaseDesigner (included in the table above in the “Other” category) is used as the standard tool VSP in patients CLP. The software is applied for precise skeletal movement planning and the design of surgical splints, which are then fabricated using 3D printing technology. Reported software functionalities include an intuitive user interface, straightforward anatomical segmentation, and smooth integration with splint design and printing workflows. Clinical outcomes are reported to be mostly consistent with the virtual plan, with postoperative skeletal positioning typically within ±2 mm of the intended movements. Some limitations remain, such as the lack of advanced soft tissue simulation and the need for manual adjustments when integrating CBCT data. VSP in orthognathic surgery for patients with isolated CLP was consistently reported to outperform traditional 2D methods in terms of accuracy, predictability, and the ability to individualize surgical approaches. Several studies have shown that up to 83% of 2D-based treatment plans required significant corrections after converting to 3D-VSP, particularly in terms of yaw rotation and midline alignment, with skeletal interferences identified in nearly half of the cases [43,53]. The use of CAD/CAM technology allows for average translational deviations within 1.6–2.3 mm and rotational deviations between 1.2 and 2.75°, supporting its clinical applicability [34,59]. Additionally, the implementation of patient-specific surgical guides and plates significantly improves the transfer accuracy of virtual plans into the operating room [60].

When it comes to the choice of surgical strategy, the included reports support the superiority of two-jaw surgery in cases with significant anatomical deformities and skeletal asymmetries. In the study by Tsai et al., cases with maxillary setbacks greater than 6 mm showed an inferior symmetry and skeletal rotation correction using single-jaw surgery, whereas bimaxillary surgery provided a more accurate midline alignment, occlusal plane leveling, and better mandibular fit [37]. Our analysis confirms these findings: two-jaw procedures offer improved aesthetic and functional outcomes, particularly in cleft patients with severe maxillary deficiency [15].

An important aspect of VSP is its integration with CAD/CAM, which streamlines the surgical workflow—from virtual planning to guide and plate fabrication—while improving plan transfer to the operating field. Despite the higher costs, these systems reduce operative time and eliminate the need for traditional wax models [61]. However, the virtual planning workflow introduces new potential sources of error, such as CBCT segmentation, intraoral scan registration, and guide positioning. All of them must be managed through clinician training and protocol standardization [62].

It is also important to note that only a limited number of studies assess long-term treatment outcomes such as skeletal stability, patient QoL, or subjective aesthetic satisfaction. Although the early results are promising, further studies—particularly randomized controlled trials—are needed to evaluate different planning and treatment strategies in terms of cost-effectiveness, patient experience, and long-term stability [53].

## 4. Discussion

This study focused on charting key characteristics of studies, technological tools, surgical approaches, LoEs, and patient-centered outcomes, rather than providing a quantitative meta-analysis.

One of the main findings was the predominance of lower quality LoEs. As summarized in the results section, LoE III and IV studies dominated, with no randomized controlled trials (LoE I) identified, and only one lower-quality RCT (LoE II) reported (Results, Section 3.1). This pattern aligns with broader trends in maxillofacial and orthognathic surgery, where case series and retrospective comparative designs remain the norm. Although this evidence provides valuable insights into current practice, the limited methodological rigor restricts the strength of inferences and underscores the need for more robust prospective cohort studies and RCTs.

Across the reviewed literature, bimaxillary orthognathic surgery (two-jaw) emerged as the preferred surgical approach in cleft patients, especially in cases of significant maxillary hypoplasia and sagittal discrepancies. Tsai et al. (2022) reported that maxillary setbacks > 6 mm were associated with inferior symmetry and rotational control when addressed with single-jaw surgery, whereas bimaxillary procedures yielded a significantly improved midline alignment and occlusal plane correction [37]. In our review, this trend is reflected by the high frequency of two-jaw procedures within the included studies, emphasizing the role of VSP in facilitating complex surgical planning for severe deformities.

The relationship between procedure type and LoE was further investigated (Figure 3). Bimaxillary OGS was the most widely published, predominantly at LoEs III and IV. Le Fort I osteotomies were primarily analyzed in studies of lower LoEs (IV and V). DO and AMSO procedures were significantly less frequently described. A noticeable scarcity of higher-level studies (LoE II) indicates the limited availability of research with strong methodological quality in this field.

Figure 4 and Figure 5 list the most frequently used VSP programs. If the authors specified a different program, it was included in the “Other” category; if no software was mentioned, the study was omitted from these visualizations. Software popularity over time was also assessed. Rhinoceros and Dolphin 3D showed the largest growth in 2019 and 2022, while the “Other” category expanded markedly from 2023 to 2025, likely reflecting a diversification of digital tools or emergence of new platforms. ProPlan CMF was the most frequently used software in 2017, whereas earlier years were characterized by a very limited output [63].

The temporal distribution of LoEs (Figure 2 and Figure 4) revealed that publications prior to 2016 were sporadic and primarily at LoE IV. From 2017 onwards, article numbers increased substantially, peaking in 2020, and continued to be dominated by LoEs III and IV. In 2023–2025, the predominance of LoE III and IV persisted, indicating a modest improvement in evidence quality, though LoE II studies remained scarce [51,64,65,66]. These observations highlight progressive clinical interest but also reinforce the critical need for more advanced study designs.

Beyond methodological classification, several papers presented clinical outcomes associated with VSP use. These included improved facial symmetry, shorter operative times, reduced intraoperative adjustments, and higher aesthetic satisfaction. The findings consistently indicated more stable skeletal corrections, reduced relapse, and higher aesthetic scores. Some studies suggested shorter surgical durations and fewer intraoperative corrections, translating to a greater procedural safety and recovery efficiency.

The accumulated data indicate that VSP significantly changes the way orthognathic treatment is planned and implemented in patients with nonsyndromic CLP, particularly in cases of complex maxillofacial deformities. The most consistently reported benefits include increased precision in jaw movement planning, better asymmetry control, and reduced intraoperative adjustments through the use of surgical splints, cutting guides, and customized plates. VSP also promotes improved communication within the multidisciplinary team and enables the more predictable planning of two-stage and bimaxillary procedures, which are common and technically demanding in the CLP population. In this regard, VSP can be considered a tool with a real impact on daily clinical practice, especially in centers with appropriate digital infrastructure.

At the same time, this scoping review highlights significant gaps in the current evidence base. The available studies address limited research on the long-term stability of outcomes, patient QoL, functional aspects, and cost-effectiveness of implementing VSP in the treatment of CLP patients. Most publications are based on observational designs with low or moderate LoEs (III–IV), which limits the ability to make firm clinical recommendations. Consequently, many of the observed benefits—such as potential improvements in postoperative stability and reduced reoperation rates—should currently be considered hypothesis-generating rather than practice-changing. These results highlight the need for well-designed prospective and randomized studies to verify the long-term clinical value of VSP in this specific patient population.

This review has several notable limitations. First, as a scoping review, its primary aim was to describe available sources rather than to critically appraise methodological quality, which restricts the ability to formulate strong clinical recommendations. Second, the included studies were characterized by a substantial heterogeneity in study design, applied virtual planning tools, and surgical procedures, which complicates direct comparisons. Third, despite a broad and systematic search strategy across multiple databases, the possibility of missing relevant publications remains. Fourth, most studies involved small sample sizes and were often retrospective, further limiting the overall strength of the evidence.

A major constraint in the current body of literature is the impediment to conducting randomized controlled trials. Future RCTs should be planned to use different VSP systems, incorporate larger patient cohorts, and include clearly defined follow-up protocols to appropriately assess long-term clinical, functional, and quality-of-life (QoL) outcomes. Such trials would allow for a more definitive evaluation of VSP’s true impact.

## 5. Conclusions

This scoping review indicates that, across the available literature, virtual surgical planning (VSP) in nonsyndromic cleft orthognathic surgery has been reported to be associated with a high planning accuracy, improved skeletal symmetry, and fewer intraoperative adjustments when compared with traditional, analog approaches. Two-jaw procedures appear to be most commonly adopted for severe maxillary hypoplasia, with several studies describing a more stable midline alignment and occlusal plane control when compared with single-jaw techniques. Regarding digital platforms, Dolphin 3D, ProPlan CMF, and several emerging systems were among the most frequently reported software solutions, although their use has evolved over time and increasingly diversified in recent years.

Overall, the current evidence suggests that VSP facilitates complex surgical planning and may be associated with clinical benefits, particularly in cases requiring extensive skeletal correction. The use of patient-specific guides and plates has also been reported to be associated with enhanced precision and reduced intraoperative modifications. However, most of the existing data originate from studies classified as LoE II–IV, and randomized or prospective investigations remain limited. These findings highlight the need for future high-quality research to validate long-term outcomes, quantify functional stability, and more clearly define the comparative advantages of virtual over conventional planning methods.

## Figures and Tables

**Table 1:** Eligibility criteria.

	Criteria for Inclusion	Criteria for Exclusion
Population	Studies involving patients with isolated CLP undergoing orthognathic evaluation or treatment using VSP.	Studies focusing exclusively on syndromic patients (e.g., Van der Woude syndrome, Treacher Collins syndrome, Pierre Robin sequence, Stickler syndrome, DiGeorge syndrome, Ehlers–Danlos, Opitz, Kabuki, Smith–Lemli–Opitz, and similar syndromes) where the craniofacial dysmorphology is not isolated CLP.
Concept	VSP applied to orthognathic surgery in non-syndromic CLP patients, regardless of software, workflow, or surgical technique.	VSP studies where the primary surgical scope extends beyond orthognathic surgery, e.g., including cranial vault, clavicles, or unrelated anatomical regions.
Context	Any clinical, surgical, or academic setting in which VSP for CLP orthognathic surgery is described.	Non-human studies, laboratory, or purely engineering simulation studies with no clinical context.
Comparators	Any or none.	Not applicable.
Outcomes	Not restricted; any reported clinical, surgical, workflow, accuracy, complication, or patient-centered outcomes eligible.	Not applicable.
Study designs	All empirical study designs assessed according to the Oxford Centre for Evidence-Based Medicine 2011 Levels of Evidence scale [21].	Not applicable.
Timeframe	No publication date limit.	Not applicable.
Language	All languages where full text is available.	None beyond inaccessibility of full text.

**Table 2:** Search engine scopes.

Engine	Website Address	Scope, Records
The ACM Guide	https://libraries.acm.org/digital-library/acm-guide-to-computing-literature (accessed on 22 July 2025)	Over 3,900,000
BASE	https://www.base-search.net (accessed on 22 July 2025)	Over 400,000,000
Cochrane	https://www.cochranelibrary.com (accessed on 22 July 2025)	Over 2,000,000
Embase	https://www.embase.com/search/quick (accessed on 22 July 2025)	Over 45,600,000
PubMed	https://pubmed.ncbi.nlm.nih.gov (accessed on 22 July 2025)	Over 38,000,000
Scholar	https://scholar.google.com (accessed on 22 July 2025)	Over 160,000,000

**Table 3:** Included reports.

First Author, Year	LoE	DOI Number	Software Used to Plan the Procedure	Complications	Follow-Up Time
Fukuyama, 2025 [25]	IV	10.1097/PRS.0000000000011728	N/A	N/A	N/A
Salinero, 2025 [26]	III	10.1097/PRS.0000000000011463	N/A	N/A	N/A
Varidel, 2025 [27]	III	10.1097/PRS.0000000000011433	Synthes TruMatch 3D printing plates, Imaging Sciences International CBCT i-CAT, Planmeca ProMax 3D Max, Fujifilm Synapse 3D V5	N/A	12 months
Beek, 2024 [28]	III	10.1007/s00784-024-05517-5	IPS Case Designer, Orthognathic Analyzer	N/A	N/A
Cáceres, 2024 [29]	IV	10.11606/D.25.2024.tde-07082024-151247	Nemotec NemoFAB + 3DMedX OGA 2.0	N/A	0.033 months
Liao, 2024 [30]	III	10.1097/PRS.0000000000011173	CBCT analysis only	N/A	N/A
Mariotto, 2024 [7]	III	10.11606/D.61.2024.tde-04102024-142112	N/A	N/A	N/A
Visser, 2024 [31]	III	10.1016/j.ijom.2023.10.353	CBCT analysis only	N/A	N/A
Merta, 2023 [32]	IV	10.1016/j.bjps.2022.10.051	Planmeca ProModel, Planmeca Romexis, 3D Systems Geomagic Freeform	One nose bleed; two VPI surgeries (Furlow) 14, 15 msc postop; two delayed wound closures	N/A
Nys, 2023 [33]	IV	10.1016/j.jormas.2023.101421	ProPlan, Amira	N/A	12 months
Bollato, 2022 [34]	III	10.3390/jcm11092675	Dolphin Imaging, DDS-Pro	N/A	N/A
Marya, 2022 [35]	V	10.2174/18742106-v16-e2202240	N/A	N/A	N/A
Matsushita, 2022 [36]	IV	10.1016/j.adoms.2022.100379	ProPlan CMF	N/A	N/A
Tsai, 2022 [37]	IV	10.3390/app12094461	Dolphin 3D, SimPlant O&O	N/A	N/A
Dibbs, 2021 [38]	V	10.1097/SCS.0000000000007711	N/A	N/A	6 months
Gerbino, 2021 [39]	IV	10.1016/j.jcms.2021.03.004	3D Slicer Geomagic Wrap	N/A	12 months
Ho, 2021 [40]	IV	10.1016/j.bjps.2021.03.030	N/A	N/A	N/A
Zhai, 2021 [41]	III	10.1097/SCS.0000000000006932	N/A	N/A	N/A
Denadai, 2020 [1]	V	10.1177/1055665620949113	CAD/CAM workflow	N/A	N/A
Denadai, 2020 [42]	V	10.1016/j.bj.2019.12.008	N/A	N/A	N/A
Hsu, 2020 [43]	III	10.1038/s41598-020-58682-4	SimPlant O&O	N/A	N/A
Seo, 2020 [44]	II	10.1097/SAP.0000000000002079	Dolphin 3D; CBCT i-CAT Dental Imaging System	N/A	N/A
Wang, 2020 [45]	III	10.1016/j.bjps.2019.07.003	Mimics 12.0	Four relapses, twelve velopharyngeal impairments, eighteen nasal changes, twelve infections	N/A
Seo, 2019 [46]	III	10.3390/jcm8122116	Dolphin 3D	Three patients with cleft and one patient without cleft presented with lip or chin numbness at 1–6 months postoperatively, with full recovery at long-term evaluations	N/A
Tsai, 2019 [47]	III	10.1016/j.jfma.2018.12.011	Rhinoceros	N/A	6 months
Wu, 2019 [48]	IV	10.1097/PRS.0000000000005646	Rhinoceros 5.0, Geomagic studio 12	N/A	6 months
Wang, 2019 [49]	IV	10.1097/SCS.0000000000005603	Mimics 17.0	0	Mean: 15 months (12–20 months)
Chang, 2017 [50]	IV	10.1097/SCS.0000000000003567	ProPlan CMF	One pseudorelapse	Mean: 23.1 months
Lai, 2017 [51]	III	PUI: L617893731	N/A	N/A	N/A
Scolozzi, 2017 [52]	V	10.1597/15-208	ProPlan CMF 2.0	0	12 months
Lo, 2016 [53]	III	PUI: L611868330	N/A	N/A	N/A
Suenaga, 2016 [54]	IV	10.1016/j.ijscr.2016.10.004	Freeform Plus	N/A	N/A
Germec-Cakan, 2014 [55]	IV	10.1016/j.ajodo.2013.06.021	Simplant OMS	N/A	12 months
Gautam, 2011 [56]	V	10.2319/070110-369.1	SIMULIA Abaqus	N/A	N/A
Minami, 2007 [57]	IV	10.1597/04-204.1	Mimics; Magics RP	N/A	N/A
Tang, 1994 [58]	IV	PMID: 16538304	N/A	N/A	N/A

N/A = Not Applicable.

**Table 4:** Major VSP software usage in orthognathic surgeries for cleft non-syndromic patients.

Software	Number of Studies	% Usage	Advantages	Disadvantages	LoE	Mean Sample Size	Average Deviation	Application Scope	Clinical Validation
Dolphin 3D	4	13.8%	Widely available, user-friendly interface, integration with CBCT and 2D images	Limited automation, lack of dynamic simulations	III–IV	26	<2 mm	Skeletal planning, splint design	Four studies (mean deviation <2 mm)
ProPlan CMF	4	13.8%	Advanced 3D modeling, compatible with 3D printing	High cost, steep learning curve	III–IV	18	<2 mm	Bimaxillary planning, STL export	Three studies (positioning accuracy >90%)
SimPlant	3	10.3%	Integration with implantology, CAD/CAM support	Low user base, limited orthodontic support	IV	12	Not reported	Implant planning, basic orthognathic use	Reported in two case series
Mimics	3	10.3%	Detailed 3D segmentation	Requires expert knowledge	IV	15	~2 mm	Segmentation, visualization	Cited in two studies
Rhinoceros	2	6.9%	Flexible modeling with open plugins	Steep learning curve	IV–V	10	Not reported	Custom design, 3D reconstruction	Not consistently reported
Other	13	44.8%	-	-	IV–V	-	-	Mixed use (STL prep, CBCT, CAD/CAM)	Scattered mentions, no unified data

## Data Availability

The original contributions presented in this study are included in the article. Further inquiries can be directed to the corresponding author.

## Supplementary Materials

The following supporting information can be downloaded at: https://www.mdpi.com/article/10.3390/jcm15020911/s1, Table S1. Risk of bias assessment—cohort studies; Table S2. Risk of bias assessment—non-randomized comparative studies; Table S3. Risk of bias assessment—analytical cross-sectional studies; Table S4. Risk of bias assessment—case series; Table S5. Risk of bias assessment—case reports and conference abstracts; Table S6. Risk of bias assessment—diagnostic test accuracy studies; Table S7. Risk of bias assessment—expert opinions (textual evidence).

## Appendix A

**Table A1:** Specific queries for each engine.

Engine	Query
The ACM Guide	[[All: orthognathic] OR [All: orthognathics] OR [All: fort]] AND [[All: cleft] OR [All: fissure] OR [All: dysjunction] OR [All: disjunction]] AND [[All: vsp] OR [All: casp] OR [All: dsw] OR [All: 3dsp] OR [All: 3d-vsp] OR [All: cad/cam] OR [All: vos] OR [All: dtp]]
BASE	(orthognathic OR orthognathics OR fort) AND (vsp OR virtual OR casp OR computer OR dsw OR digital OR workflow OR 3dsp OR 3d-vsp OR cad OR cam OR vos OR dtp OR proplan OR planmeca OR romexis OR dolphin) AND (fissure OR split OR groove OR notch OR slit OR cleft OR dysjunction OR disjunction)
Cochrane	(orthognathic OR orthognathics OR fort) AND (vsp OR virtual OR casp OR computer OR dsw OR digital OR workflow OR 3dsp OR 3d-vsp OR cad OR cam OR vos OR dtp OR proplan OR planmeca OR romexis OR dolphin) AND (fissure OR split OR groove OR notch OR slit OR cleft OR dysjunction OR disjunction)
Embase	(orthognathic OR orthognathics OR fort) AND (cleft OR ‘fissure’/exp OR fissure OR dysjunction OR ‘disjunction’/exp OR disjunction) AND (‘vsp’/exp OR vsp OR casp OR dsw OR 3dsp OR ‘3d vsp’ OR ‘cad’/exp OR cad OR dtp)
PubMed	(orthognathic OR orthognathics OR fort) AND (vsp OR virtual OR casp OR computer OR dsw OR digital OR workflow OR 3dsp OR 3d-vsp OR cad OR cam OR vos OR dtp OR proplan OR planmeca OR romexis OR dolphin) AND (fissure OR split OR groove OR notch OR slit OR cleft OR dysjunction OR disjunction)
Scholar	allintitle: (orthognathic OR orthognathics OR fort) AND (vsp OR virtual OR casp OR computer OR dsw OR digital OR workflow OR 3dsp OR 3d-vsp OR cad OR cam OR vos OR dtp OR proplan OR planmeca OR romexis OR dolphin) AND (fissure OR split OR groove OR notch OR slit OR cleft OR dysjunction OR disjunction)
