# Supplementary material for: Virtual Surgical Planning (VSP) in Orthognathic Surgery for Non-Syndromic Cleft Patients: A Scoping Review of Trends and Clinical Outcomes
Source: J Clin Med. 2026 Jan 22;15(2):911. doi: 10.3390/jcm15020911 (PMC12841811; doi:10.3390/jcm15020911)
Supplement: Supplementary file 1 [file jcm-15-00911-s001.zip › jcm-4067724-supplementary.pdf]

Table S1. Risk of bias assessment - cohort studies

**First author, year**	**Paper type**	**DOI**	**Were the two groups similar and recruited from the same population?**	**Were the exposures measured similarly to assign people to both exposed and unexposed groups?**	**Was the exposure measured in a valid and reliable way?**	**Were confounding factors identified?**	**Were strategies to deal with confounding factors stated?**	**Were the groups/participants free of the outcome at the start of the study (or at the moment of exposure)?**	**Were the outcomes measured in a valid and reliable way?**	**Was the follow up time reported and sufficient to belong enough for outcomes to occur?**	**Was follow up complete, and if not, were the reasons to loss to follow up described and explored?**	**Were strategies to address incomplete follow up utilized?**	**Was appropriate statistical analysis used?**	**Risk of bias**

Fukuyama, 2025 [25]	Cohort study	10.1097/PRS.00000000000011728	Yes	Yes	Yes	Yes	Unclear	Yes	Yes	Unclear	Unclear	Unclear	Yes	Moderate
Salinero, 2025 [26]	Cohort study	10.1097/PRS.00000000000011463	Yes	Yes	Yes	Unclear	Unclear	Unclear	Yes	No	No	No	Yes	Moderate
Liao, 2024 [30]	Cohort study	10.1097/PRS.00000000000011173	Yes	No	Yes	Unclear	Unclear	Yes	Yes	Unclear	Unclear	Unclear	Yes	Moderate
Visser, 2024 [31]	Cohort Study	10.1016/j.ijom.2023.10.353	Yes	Yes	Yes	Yes	Yes	Yes	Yes	Unclear	Yes	Yes	Yes	Low
Bollato, 2022 [34]	Cohort study	10.3390/jcm11092675	Yes	Yes	Yes	No	Unclear	Yes	Yes	Unclear	Yes	Yes	Yes	Low
Gerbino, 2021 [39]	Cohort study	10.1016/j.jcms.2021.03.004	Unclear	Yes	Yes	Unclear	No	Yes	Yes	Yes	Unclear	No	Yes	Moderate
Ho, 2021 [40]	Cohort study	10.1016/j.bjps.2021.03.030	Yes	Yes	Yes	Unclear	Unclear	Yes	Yes	Unclear	Unclear	No	Yes	Moderate
Zhai, 2021 [41]	Cohort study	10.1097/SCS.00000	Unclear	Unclear	Yes	Unclear	No	Yes	Yes	Unclear	Unclear	No	Yes	Moderate

		00000006932											
Wang, 2020 [45]	Cohort study	10.1016/j.bjps.2019.07.003	Unclear	Yes	Yes	Unclear	No	Yes	Yes	Yes	Yes	Yes	Yes	Moderate
Seo, 2019 [46]	Cohort study	10.3390/jcm8122116	Yes	Yes	Yes	Unclear	Unclear	Yes	Yes	Unclear	Yes	No	Yes	Moderate
Wu, 2019 [48]	Cohort study	10.1097/PRS.00000000000005646	Yes	Yes	Yes	Unclear	Unclear	Yes	Yes	Unclear	Yes	No	Yes	Moderate
Lo, 2016 [53]	Cohort study	PUI: L611868330	Yes	Yes	Yes	Partial	Partial	Yes	Yes	Yes	Yes	Partial	Yes	Low
Gautam, 2011 [56]	Finite element (laboratory/in silico study)	10.2319/070110-369.1												JBI does not provide a formal assessment tool for finite element analysis (FEA), so it is not officially assessed

														in any of these categories .
Hsu, 2020 [58]	Cohort Study	10.1038/s41598-020-58682-4	Yes	Yes	Yes	Unclear	Unclear	Yes	Yes	Yes	Unclear	No	Yes	Moderate
Seo, 2020 [44]	Cohort Study	10.1097/SAP.0000000000002079	Yes	Yes	Yes	Unclear	No	Yes	Yes	Yes	Unclear	No	Yes	Moderate

Table S2. Risk of bias assessment - non-randomized comparative studies.

First author, year	Paper type	DOI	Is it clear in the study what is the 'cause' and what is the 'effect' (i.e. there is no confusion about which variable comes first)?	Were the participants included in any comparisons similar?	Were the participants included in any comparisons receiving similar treatment/care, other than the exposure or intervention of interest?	Was there a control group?	Were there multiple measurements of the outcome both pre and post the intervention/exposure?	Was follow up complete and, if not, were differences between groups in terms of their follow up adequately described and analyzed?	Were the outcomes of participants included in any comparisons measured in the same way?	Were outcomes measured in a reliable way?	Was appropriate statistical analysis used?	Risk of bias
Varidel, 2025 [27]	Quasi-Experimental Study (non-randomized comparative design)	10.1097/PRS.0000000000011433	Yes	Yes	Yes	Yes	Unclear	Unclear	Yes	Yes	Yes	Low to Moderate

Cáceres, 2024 [29]	Quasi-Experimental Study (non-randomized comparative design)	10.11606/D.25.2024.tde-07082024-151247	Yes	Unclear	Yes	No	Yes	Unclear	Yes	Yes	Yes	Moderate

Table S3. Risk of bias assessment - analytical cross-sectional studies.

First author, year	Paper type	DOI	1. Were the criteria for inclusion in the sample clearly defined?	2. Were the study subjects and the setting described in detail?	3. Was the exposure measured in a valid and reliable way?	4. Were objective, standard criteria used for measurement of the condition?	5. Were confounding factors identified?	6. Were strategies to deal with confounding factors stated?	7. Were the outcomes measured in a valid and reliable way?	8. Was appropriate statistical analysis used?	Risk of bias
Beek, 2024 [28]	Analytical Cross-Sectional Study	10.1007/s00784-024-05517-5	Yes	Yes	Yes	Yes	Unclear	Unclear	Yes	Yes	Low to Moderate
Merta, 2023 [32]	Analytical Cross-Sectional Study	10.1016/j.bjps.2022.10.051	Yes	Yes	Yes	Yes	Unclear	No	Yes	Unclear	Moderate to High
Nys, 2023 [33]	Analytical Cross-Sectional Study	10.1016/j.jormas.2023.101421	Yes	Yes	Yes	Yes	Unclear	No	Yes	Unclear	Moderate to High
Tsai, 2022 [37]	Analytical Cross-Sectional Study	10.3390/app12094461	Yes	Yes	Yes	Yes	Unclear	No	Yes	Yes	Moderate
Tsai, 2019 [47]	Analytical Cross-Sectional Study	10.1016/j.jfma.2018.12.011	Yes	Yes	Yes	Yes	Yes	Unclear	Yes	Yes	Low to Moderate

Table S4. Risk of bias assessment - case series.

First author, year	Paper type	DOI	Were there clear criteria for inclusion in the case series?	Was the condition measured in a standard, reliable way for all participants included in the case series?	Were valid methods used for identification of the condition for all participants included in the case series?	Did the case series have consecutive inclusion of participants?	Did the case series have complete inclusion of participants?	Was there clear reporting of the demographics of the participants in the study?	Was there clear reporting of clinical information of the participants?	Were the outcomes or follow-up results of cases clearly reported?	Was there clear reporting of the presenting site(s)/clinic(s) demographic information?	Was appropriate statistical analysis used?	Risk of bias
Dibbs, 2021 [38]	Case Series	10.1097/SCS.00000000000007711	Yes	Yes	Yes	Unclear	Unclear	Yes	Yes	Yes	Yes	No	Low to Moderate
Denadai, 2020 [1]	Case Series	10.1177/1055665620949113	Yes	Yes	Yes	Unclear	Unclear	Yes	Yes	Yes	Yes	No	Low to Moderate
Wang, 2019 [49]	Case series	10.1097/SCS.00000000000005603	Unclear	Yes	Yes	Unclear	No	Yes	Yes	Unclear	Yes	Yes	Moderate
Chang, 2017 [50]	Case Series	10.1097/SCS.00000000000003567	Yes	Yes	Yes	Unclear	Unclear	Yes	Yes	Yes	Yes	No	Low to Moderate
Minami, 2007 [57]	Case Series	10.1597/04-204.1	Yes	Yes	Yes	Unclear	Unclear	Yes	Yes	Yes	Yes	No	Low to Moderate
Tang, 1994 [58]	Case Series	PMID: 16538304	Yes	Yes	Yes	Unclear	Unclear	Yes	Yes	Yes	Yes	No	Low to Moderate
Matsushita, 2022 [36]	Case Series	10.1016/j.adoms.2022.100379	Yes	Yes	Yes	Unclear	Unclear	Yes	Yes	Yes	Yes	No	Low to Moderate
Scolozzi, 2007 [59]	Case Series	10.1597/15-208	Yes	Yes	Yes	Unclear	Unclear	Yes	Yes	Yes	Yes	No	Low to Moderate

Table S5. Risk of bias assessment - case reports and conference abstracts.

First author, year	Paper type	DOI	Were patient's demographic characteristics clearly described?	Was the patient's history clearly described and presented as a timeline?	Was the current clinical condition of the patient on presentation clearly described?	4. Were diagnostic tests or assessment methods and the results clearly described?	Was the intervention(s) or treatment procedure(s) clearly described?	Was the post-intervention clinical condition clearly described?	Were adverse events (harms) or unanticipated events identified and described?	Does the case report provide takeaway lessons?	Risk of bias
Denadai, 2020 [60]	Case Report	10.1016/j.bj.2019.12.008	Yes	Yes	Yes	Yes	Yes	Yes	No	Yes	Low
Lai, 2017 [51]	Conference Abstract (Not Appraised)	PUI: L617893731									JBI does not provide a formal assessment tool for conference papers.
Suenaga, 2016 [54]	Case Report	10.1016/j.ijscr.2016.10.004	Yes	Yes	Yes	Yes	Yes	Yes	No	Yes	Low
Germec-Cakan, 2014 [55]	Case Report	10.1016/j.ajodo.2013.06.021	Yes	Yes	Yes	Yes	Yes	Yes	No	Yes	Low

Table S6. Risk of bias assessment - diagnostic test accuracy studies.

First author, year	Paper type	DOI	Was a consecutive or random sample of patients enrolled?	Was a case control design avoided?	Did the study avoid inappropriate exclusions?	Were the index test results interpreted without knowledge of the results of the reference standard?	If a threshold was used, was it pre-specified?	Is the reference standard likely to correctly classify the target condition?	Were the reference standard results interpreted without knowledge of the results of the index test?	Was there an appropriate interval between index test and reference standard?	Did all patients receive the same reference standard?	Were all patients included in the analysis?	Risk of bias
Mariotto, 2024 [7]	Diagnostic Test Accuracy Study	10.11606/D.61.2024.tde-04102024-142112	Unclear	Yes	Yes	Unclear	Yes	Yes	Unclear	Yes	Yes	Yes	Low to Moderate

Table S7. Risk of bias assessment - expert opinions (textual evidence).

First author, year	Paper type	DOI	Is the source of opinion clearly identified?	Does the source of opinion have standing in the field of expertise?	Are the interests of the relevant population central to the opinion?	Is the opinion's basis in logic and reasoning clearly stated?	Is there reference to the extant literature?	Are any incongruencies with the literature logically defended?	Risk of bias
Marya, 2022 [35]	Textual Evidence - Expert Opinion	10.2174/18742106-v16-e2202240	Yes	Yes	Yes	Yes	Yes	Unclear	Low
